# Treatment of Abortion*Read before the Richmond Academy of Medicine and Surgery, February 25th, 1896.

**Published:** 1896-06

**Authors:** J. W. Long

**Affiliations:** Professor of Diseases of Women and Children, Medical College of Virginia, Richmond, Virginia


### TREATMENT OF ABORTION.*

By J. W. LONG, M. D.,

Professor of Diseases of Women and Children, Medical College of Virginia, Richmond, Virginia.

A consideration of this subject involves many interesting questions. Of these I will discuss but a few, and briefly:

1. *When is abortion inevitable?*

This question settled, the course to be pursued will be clear. Uterine pains and hemorrhages have been considered as sure evidences that abortion will occur. That this is not always true, is easily proven. I recall one case that bled from the first till after the third month (with recurring pains), that went to term. Scanzoni reports a case in which occurred profuse hemorrhages in the third month, and in spite of ergot, the tampon, use of the uterine sound, and an intra-uterine injection of solution of perchloride of iron, the case went on to term. Noble† was called to a case that resisted the repeated applications of pure carbolic acid to the endometrium. Better indications that abortion is inevitable, are dilatation of the *os*, and descent of the *ovum*. Escape of the liquor amnii, or septic infection, may be considered a positive proof that the expulsion of the fœtus is inevitable.

2. *Treatment when abortion is not inevitable.*

Rest, quiet and opium are the sovereign remedies. A friend of mine kept a patient under the influence of opium the entire last seven months of her pregnancy. Pains would begin whenever the drug was discontinued, nor did she become an opium *habitue*.

3. *Inevitable abortion.*

The plain indication here is to empty the uterus, which, according to the classic maxim, is not complete until the fœtus, placenta, membranes and all clots are removed. Many times Nature herself is able to do this, and only when she fails are we to interfere.

By far the best instrument for removing the products of

---

*Read before the Richmond Academy of Medicine and Surgery, February 25th, 1896.

†A Consideration of Certain Doubtful Points in the Management of Abortion. Dr. Chas. P Noble.

conception is the finger, aided by the other hand depressing and steadying the fundus from above. In those cases where the cervix is not patulous, it becomes necessary to dilate the cervix with a steel dilator. Then the cavity is to be explored by the finger or curette, followed by a copious irrigation, and a pack of iodoform gauze.

Sometimes one is in doubt as to whether or not the uterus is empty. Especially is this true of abortions in the third and fourth months. The *ovum* passed intact, is evidence that the uterus is empty. A patulous cervix and continued bleeding indicates that something yet remains in the uterus.

*4. Septic abortion.*

In cases of this kind, delay is inexcusable. Many deaths may be put down to the credit of the let alone, waiting, non-interference policy. The products of conception broken up and infected as in criminal abortion, present the ideal condition for producing general sepsis. Not only should the product of conception be removed, but also, the infected maternal membranes. The gaping lymphatics and open Fallopian tubes will continue to spread the infection as long as any remains in the uterus. Therefore, the necessity for a thorough cleansing. In most instances this can be done only by the curette. In more advanced cases, the finger is a safe guide, because sentient. Thorough irrigation and packing are also essential.

I have not stated that all operative or explorative procedure in the uterus, must be done under strict antiseptic precautions, as this goes without saying and is hardly pertinent to the question under consideration.

I doubt that it is justifiable to curette more than once in these cases; indeed, I question whether or not uterine irrigations should be continued longer than one or two days, and it is quite probable that in those cases, where the local inflammation involves the peri-uterine to a marked degree, the manipulations necessary to intra-uterine irrigation do more harm than good.

*5. When shall further operative procedures be instituted?*

*Hysterotomy* for puerperal sepsis, is one of the burning questions of the day. The advocates for it have not yet made out their case, but have established just claims to be heard. The most that can be said, at this writing, for the operation, is that hysterotomy is indicated in those cases of sepsis where thorough irrigation and curettage have failed to modify or abase the general sepsis, and *the local septic inflammation is confined to the uterus.* I emphasize the words in italics, for thereupon will depend the result. Two such cases were reported by Dr. Cartledge, of Louisville, at the Washington meeting of the Southern Surgical and Gynecological Association, the uterus in each case being honeycombed with septic abscesses.

*Vaginal incision and drainage*, is an operation of great value in just those cases where hysterotomy is contra-indicated.

I have described the indications and technique in a paper read by title before the Southern Surgical and Gynecological Association, at the Washington meeting, in November, 1895. By this method, the sodden, septic pelvic tissues are drained of the sepsis, and the general system is also relieved.

---

"THE ownership of the prescription has been settled by law in New York, Massachusetts and a few other States. They all give the prescription to the druggist. Some time ago a judge of a court in Berlin, Germany, gave a similar decision. The text of the decision from the judge of the Supreme Court of one of our States is as follows: The question before the court seems to be very simple, indeed. A patient applies to a physician and receives from him certain advice, for which he tenders a fee. The physician hands a piece of paper to the patient, purporting to be a written order for certain goods called drugs, which order is filled by a merchant or apothecary. The payment of the fee and the delivery of the goods or drugs terminates the verbal contract, and the druggist keeps the prescription as an evidence that the contract has been fulfilled, as far as he is concerned. The druggist can, if he so please, on his own responsibility, renew the drugs, for he is but a merchant, and has a perfect right to sell drugs to any one in any shape. He need not keep the prescription, nor is he bound to give a copy, but should error occur, he has no protection in case of suit."—*Albany Medical Annals.*

